# A Novel Spatter Detection Algorithm for Real-Time Quality Control in Laser-Directed Energy Deposition-Based Additive Manufacturing

**DOI:** 10.3390/s25123610

**Published:** 2025-06-08

**Authors:** Farzaneh Kaji, Jinoop Arackal Narayanan, Mark Zimny, Ehsan Toyserkani

**Affiliations:** 1 Multi-Scale Additive Manufacturing Laboratory, Department of Mechanical and Mechatronics Engineering, University of Waterloo, Waterloo, ON N2L 3G1, Canada; ehsan.toyserkani@uwaterloo.ca; 2 School of Computing, Engineering and Digital Technologies, Teesside University, Middlesbrough TS1 3BX, UK; j.arackalnarayanan@tees.ac.uk; 3 Promation, 2767 Brighton Rd, Oakville, ON L6H 6J4, Canada; mark@promation.com

**Keywords:** laser-directed energy deposition, spatter, process monitoring, image processing, defects

## Abstract

Laser-Directed Energy Deposition (LDED) has recently been widely used for 3D-printing metal components and repairing high-value parts. One key performance indicator of the LDED process is represented by melt pool stability and spatter behavior. In this research study, an off-axis vision monitoring system is employed to characterize spatter formation based on different anomalies in the process. This study utilizes a 1 kW fiber laser-based LDED system equipped with a monochrome high-dynamic-range (HDR) vision camera and an SP700 Near-IR/UV Block visible bandpass filter positioned at various locations. To extract meaningful features from the original images, a novel image processing algorithm is developed to quantify spatter counts, orientation, area, and distance from the melt pool under harsh conditions. Additionally, this study analyzes the average number of spatters for different laser power settings, revealing a strong positive correlation. Validation experiments confirm over 93% detection accuracy, underscoring the robustness of the image processing pipeline. Furthermore, spatter detection is employed to assess the impact of spatter formation on deposition continuity. This research study provides a method for detecting spatters, correlating them with LDED process parameters, and predicting deposit quality.

## 1. Introduction

Laser-Directed Energy Deposition (LDED) has been widely used in recent years for 3D-printing metal components and repairing high-value parts [1,2]. LDED offers shape and material design freedom, enabling the fabrication of geometrically complex structures with multiple materials in a single component. However, the process faces several challenges, including geometrical instability, porosity, cracking, and residual stresses. As this technology advances to produce functional parts, effective process monitoring and control are essential to ensuring its feasibility and repeatability. A fundamental factor influencing these challenges is the stability of the melt pool. One key performance indicator of melt pool stability in the LDED process is represented by spatter formation and behavior.

**Figure 1:**
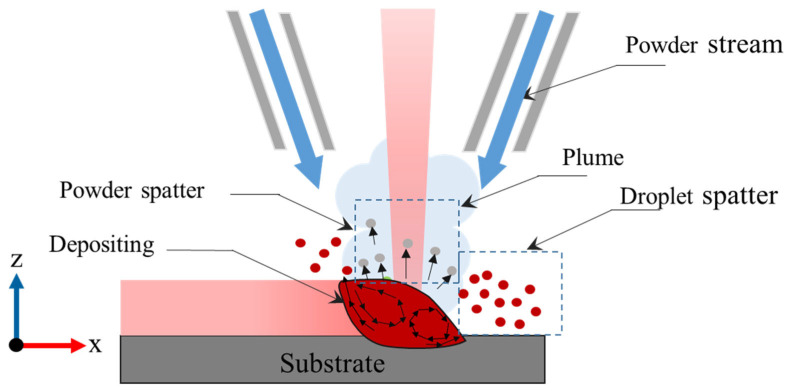
Spatter formation during LDED-PF process.

Spatter refers to the material ejected from the molten pool. In LDED, spatter can originate from unstable melt pools caused by vapor recoil pressure and Marangoni effects [3]. Strong temperature gradients in the melt pool induce temperature-dependent surface tension, leading to Marangoni effects. The resulting circulation of the liquid melt pool can generate spatter in LDED [4]. This drives the melt flow from the hot laser spot toward the cooler rear, creating the recirculation of material in the melt pool. As a result, spattering occurs when low-viscosity liquid metal is ejected from the surface, as shown in Figure 1. Another source of spatter formation in LDED occurs when powder particles in the powder stream fail to enter the melt pool. Instead, they bounce back, primarily from the denudation zone surrounding the melt pool [5,6].

In recent years, various monitoring techniques based on melt pool radiation have been applied to assess the LDED process. A melt pool monitoring system incorporating three CCD cameras was developed to detect clad height under different process conditions by using a novel image processing algorithm [7]. The formation of spatter has been extensively studied for quality assurance in both traditional and laser welding techniques. In automatic welding, spatter is commonly used as a performance and quality indicator [8]. Bidanda et al. [9] developed a spatter index as a quality indicator for the laser welding process, considering spatter area, length, and distance from the weld axis. Based on the spatter index, a decision was made to pass or fail the weld. Hugger et al. [10] experimentally investigated the effects of welding speed, laser power, and pulse frequency on spatter formation in laser welding. Using two high-speed cameras (100 kHz) and reconstruction algorithms, they determined spatter trajectories, which were then used to calculate spatter velocity. Liu et al. [11] identified two types of spatters in the Selective Laser Melting (SLM) process: droplet spatter and sideways spatter. Both types form due to the recoil pressure generated by the metallic powder. The study examined spatter characteristics and analyzed their impact on the quality of SLM components. Yin et al. [12] studied the effects of spatter and its ejection angle on the metallurgical properties of LPBF parts. Grünewald et al. [13] investigated the influence of ring-shaped beams on spatter characteristics in laser-based powder bed fusion, demonstrating that the modified beam shape affects melt pool dynamics and spatter behavior. Cui et al. [14] showed that optimizing laser power and powder feed rate can minimize spatter in LDED. Their study revealed that adjustments to the deposition parameters reduce material loss and improve the quality of coatings and printed components. Zhang et al. [15] employed an image processing algorithm to extract melt pool features, spatter characteristics, and plume behavior in the laser powder bed fusion (LPBF) process. Local thresholding methods were used, with the plume and melt pool areas masked to isolate spatter features. A Kalman filter approach was applied to predict spatter positions in subsequent frames and calculate spatter velocities. Spatter areas were analyzed in four different irregular cases as indicators of process instability. Schweier et al. [16] utilized a Gaussian blurring technique to minimize plume effects in laser welding image processing for spatter recognition. By subtracting a Gaussian blur filter from the original image, they successfully eliminated the plume. The processing zone was masked, and spatter width and velocity were analyzed to determine whether spatter originated from ablation, re-entry into the melt pool, or melt pool dynamics. Nicolosi et al. [17] also employed local thresholding and Gaussian filters for image preprocessing in spatter detection. The processing zone was automatically masked, and the impact of spatter on the top layer of laser beam welding seams was studied. Haubold et al. [18] applied Contrast-Limited Adaptive Histogram Equalization (CLAHE) [19] to enhance image contrast in the laser welding process. A Gaussian blur of the images was subtracted from the original images, followed by thresholding with a constant value, and the processing zone was masked. The algorithm was validated by generating artificial spatter images and inputting them into the system. This approach also successfully identified overlapping spatters.

Despite significant advancements in understanding spatter formation and mitigation across various laser-based manufacturing processes, a gap remains in directly correlating spatter characteristics with process parameters in LDED. Existing studies have primarily focused on laser welding and powder bed fusion techniques, with limited emphasis on real-time monitoring and predictive modeling for LDED. Notably, recent synchrotron-based studies [20,21] have explored pore evolution and partially melted powders in LDED, emphasizing the need for scalable in situ solutions. Additionally, while image processing techniques have been employed for spatter detection, a comprehensive approach that integrates real-time data acquisition with an advanced image processing pipeline to quantify spatter behavior is still lacking. Addressing these gaps could enhance process control, reduce material loss, and improve part quality in LDED applications. In this research study, the relationship between spatter characteristics and process parameters is investigated. First, an off-axis camera is used to monitor the melt pool and the generated spatter. Second, a state-of-the-art image processing algorithm is employed to extract spatter characteristics from the acquired images. Finally, correlation analysis is conducted to establish links between spatter behavior and process parameters.

## 2. Materials and Methods

SS 316L powder with a particle size range of 15–45 μm and a spherical morphology (D10 = 19.66 μm, D50 = 29.54 μm, and D90 = 44.08 μm) was used as the feedstock material. The powder was produced by Carpenter Additive (Philadelphia, PA, USA) through gas atomization.

**Figure 2:**
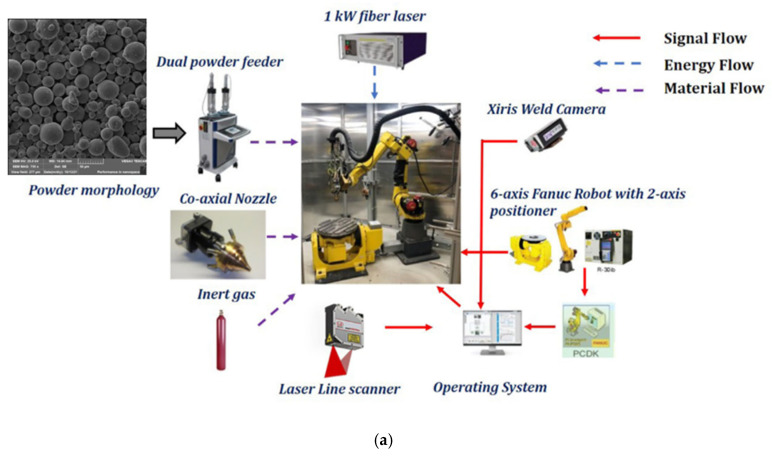

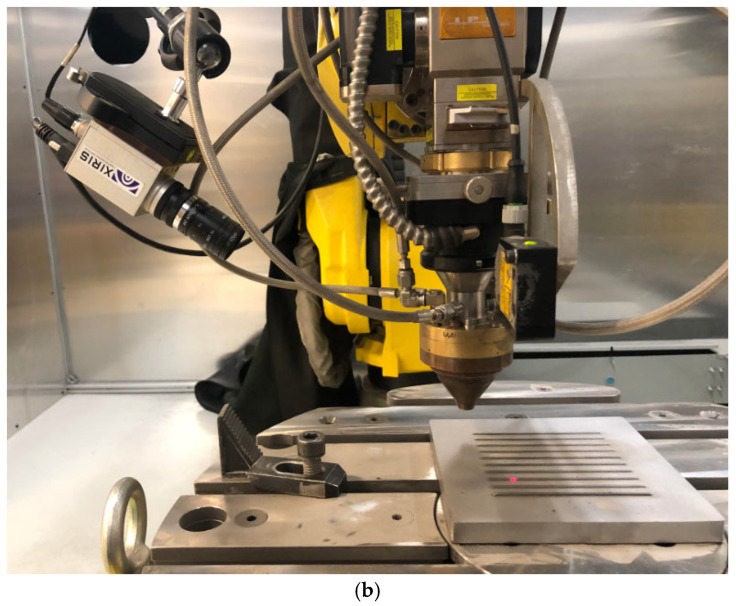
(**a**) LDEDP-PF system. (**b**) Camera orientation.

The experiments were conducted by using an in-house-developed 1 kW fiber laser-based robotic LDED system equipped with a dual-hopper powder feeder and a coaxial nozzle, as shown in Figure 2a. Melt pool and spatter monitoring were performed by using a monochrome XVC-1000 high-dynamic-range (HDR) camera with a frame rate of 60 fps and a field of view of approximately 12 × 9 mm^2^ (Xiris Automation, Burlington, ON, Canada). The camera was mounted on a bracket attached to the end effector, as shown in Figure 2b. Positioned with a leading-direction view, it captured the melt pool’s length and width along with the dimensions of the ejected spatter. A 75 mm lens with a 40 mm spacer was used to achieve a close-up view of the melt pool and spatter. A green filter was applied to detect wavelengths between 470 and 600 nm, while a UV filter was used to eliminate reflections in the region of interest. The experiments were conducted at 60 frames per second with a 5 ms exposure time [22].

Single-track geometries were deposited at varying laser power (*P*) levels while keeping the scanning speed (*v*), powder feed rate, and shielding gas feed rate constant, as shown in Table 1. The laser energy per unit length (*P*/*v*) ranged from 66 to 166 J/mm for the investigation.

## 3. Results and Discussion

### 3.1. Algorithm Development

**Figure 3:**
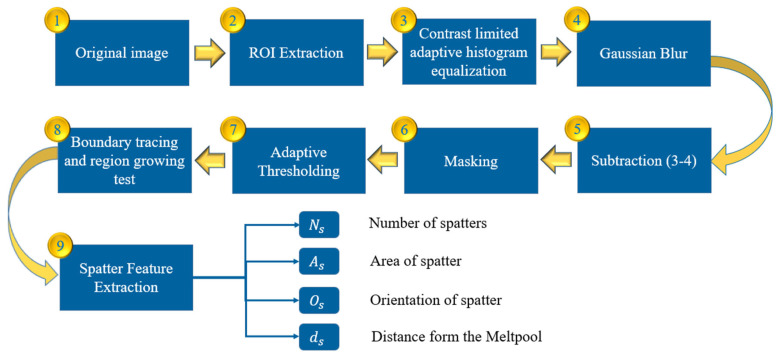
Image processing pipeline for spatter characteristic detection.

Effectively extracting valuable features from original images requires a robust image processing method. Existing image processing techniques developed for laser welding and SLM often focus on feature extraction from elements such as melt pools, plumes, and spatter [23,24]. However, these methods are not directly applicable to LDED, and our work presents a novel adaptation combining CLAHE preprocessing, Gaussian subtraction, and blob-based geometric classification under spatter-rich conditions, as the feedstock is introduced directly onto the substrate. In contrast, in SLM, the feedstock is applied atop the substrate, while in laser welding, the feedstock serves as an electrode. Therefore, when developing image processing methods for LDED, it is essential to account for the particles in the powder stream. The schematic of the image processing workflow is presented in Figure 3. First, the region of interest (ROI) is extracted from the original image. Next, a Contrast-Limited Adaptive Histogram Equalization (CLAHE) filter is applied to enhance the contrast between dark and bright regions. CLAHE is particularly useful when an image contains areas that are significantly lighter or darker than the rest, ensuring improved contrast and feature visibility [25]. The Clip Limit of 0.5 was selected to avoid the over-amplification of noise in the homogenous regions of the image. The tile grid size for the CLAHE operation was set to 50, ensuring a quadratic structure with an edge size of 50 pixels. Next, to eliminate the vapor plume from the image, a Gaussian-blurred copy of the image is subtracted from the original. This process removes smooth shading effects (e.g., the vapor plume) while preserving sharper, well-defined regions such as spatter. The following parameters for the Gaussian blur filter are proven to be robust in the segmentation of spatters and removing the vapor plume:

- Gaussian kernel standard deviation: $\sigma_{x}=\sigma_{y}=30$;
- Gaussian kernel size: k=3×3.

**Figure 4:**
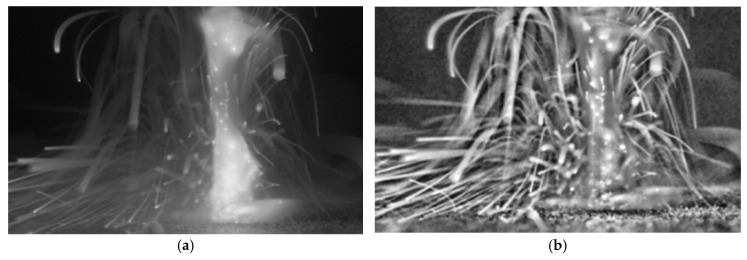
Example of image frame: (**a**) original image after ROI extraction and (**b**) image after applying CLAHE with $C_{L}=0.5$.

**Figure 5:**
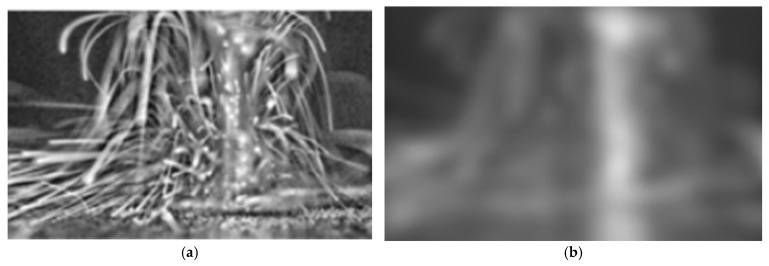
Example of image frame: (**a**) before the Gaussian blue filter and (**b**) after applying the Gaussian blur filter.

The output of the CLAHE operations is fed to the Gaussian blur as shown in Figure 3. The original image after ROI extraction and the result after applying CLAHE are shown in Figure 4a,b, respectively. Figure 5 presents the image frame before and after applying the Gaussian blue filter.

**Figure 6:**
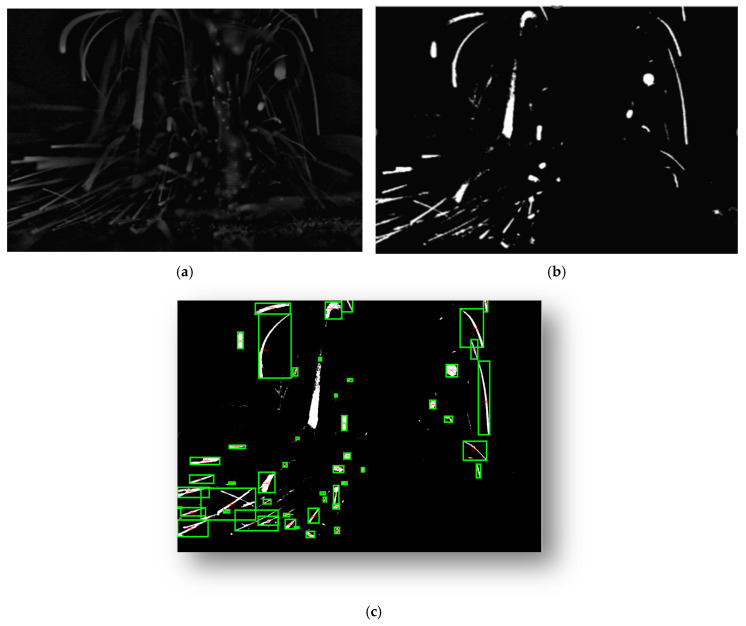
(**a**) Output of subtraction of Gaussian blue from CLAHE. (**b**) Adaptive thresholding. (**c**) Extraction of connected regions.

Once the Gaussian blur of the image is generated, it is subtracted from the output of the CLAHE process, as shown in Figure 6a. Subsequently, an adaptive thresholding technique is employed to convert the grayscale image to a binary image, as shown in Figure 6b, to separate the background and foreground of the image. Since spatters cannot be robustly separated from other artefacts in the processing zone, this region is masked out by using a custom-size rectangle mask. Finally, blob analysis is used to find the connected pixels in the resulting image given the maximum and minimum sizes of the corresponding blobs, as shown in Figure 6c. The final image (Figure 6c) only shows the connected regions that are found by using the Blob Analysis function in OpenCV. This function provides the area of the blobs, their centroids, and their bounding boxes.

### 3.2. Spatter Detection and Analysis

**Figure 7:**
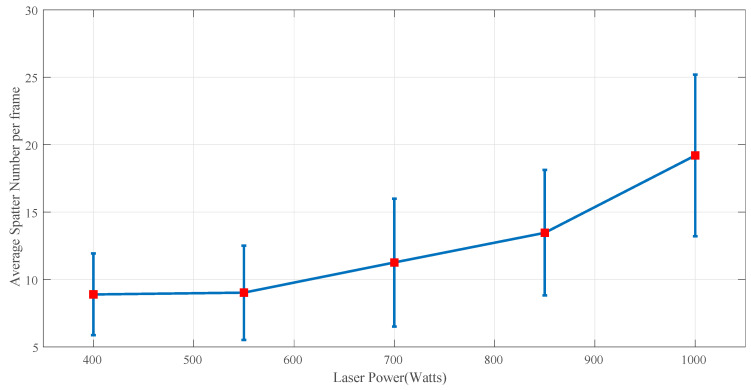
Relationship between average spatter numbers per frame and laser power.

Figure 7 illustrates the relationship between the average number of spatters and the average laser power. Higher laser power results in a larger melt pool due to increased energy input, leading to greater material melting. Since larger melt pools are inherently less stable and more susceptible to disturbances (such as surface tension variations and recoil pressure), this instability contributes to higher spatter ejection.

**Figure 8:**
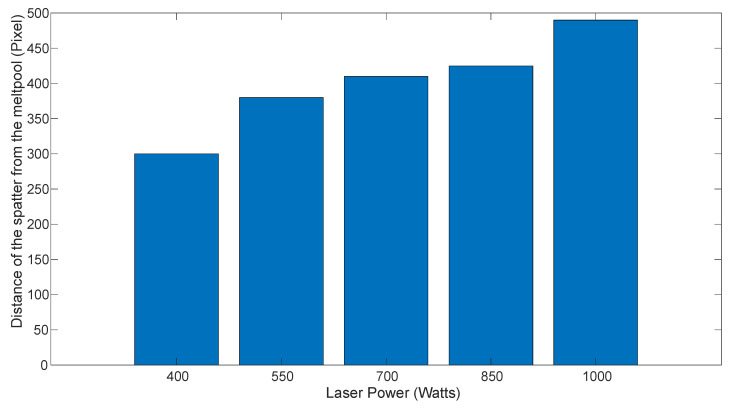
Relationship of the distance of spatter from the melt pool and the laser power.

Figure 8 illustrates the relationship between spatter distance from the melt pool and average laser power. The results suggest that spatter velocity increases with higher laser power. As laser energy input rises, more intense recoil pressure and vapor plume effects eject molten material at higher velocities, increasing the distance of spatters from the melt pool. Additionally, stronger thermal gradients and Marangoni convection enhance melt pool dynamics, further propelling spatter particles away. The greater kinetic energy imparted to the spatter, resulting from intensified vaporization and increased melt pool instability, contributes to the observed increase in spatter distance.

**Figure 9:**
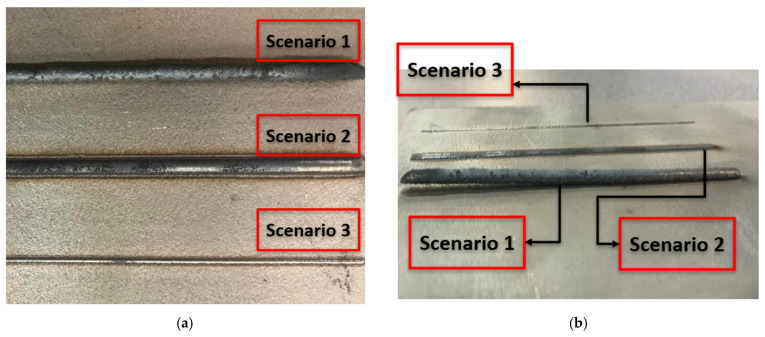
Effect of different process parameters on clad properties. (**a**) Top view and (**b**) side view.

To assess the impact of spatter on the quality of LDED components and the deposition process, multiple experimental scenarios were analyzed to establish correlations between process irregularities and spatter characteristics. In Scenario 1, process parameters were intentionally selected to induce delamination between the deposited bead and the substrate. In contrast, Scenarios 2 and 3 utilized parameters from a stable process window to ensure consistent deposition conditions, as shown in Figure 9.

**Figure 10:**
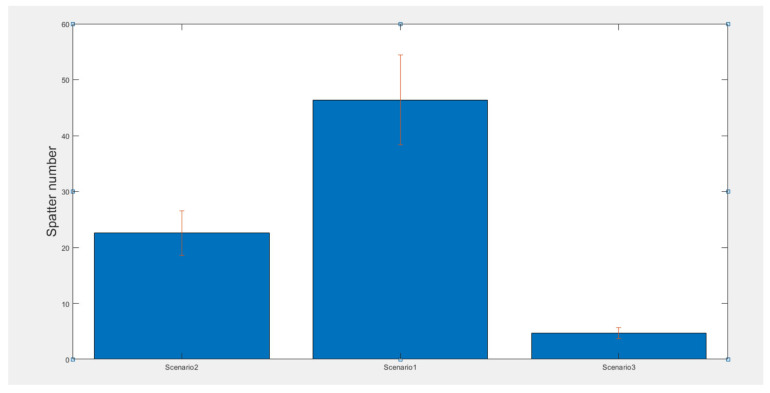
The spatter number for a single-track build in different scenarios.

To investigate the correlation between process irregularities and spatter characteristics, the number of spatters was recorded during each single-track deposition process. The analysis of the average spatter count per frame for each deposition process revealed that spatter generation increased during irregular deposition with unstable process parameters, as shown in Figure 10. This trend suggests that the spatter count per frame could serve as a potential indicator for detecting process instability.

### 3.3. Validation and Accuracy

A subset of the video frames was manually annotated to create a ground-truth dataset. The algorithm achieved 93.4% average precision in detecting spatter events, demonstrating robust performance across different laser powers. Detection results for representative frames are shown in Figure 5, with overlays of identified spatter. These metrics support the effectiveness of the proposed method in realistic deposition environments. To further validate the reliability of the measurements, an error analysis was conducted by calculating the standard deviation and coefficient of variation for spatter counts across repeated trials under the same process parameters. The standard deviation remained below 12% of the mean spatter count, confirming the algorithm’s consistency. Dispersion ranges were also computed to assess variability in detected spatter size and spatial distribution, which remained stable across the dataset.

### 3.4. Sensitivity Analysis

The algorithm was tested under simulated lighting variations and exposure settings. Performance metrics deviated less than 5% from the baseline, confirming the resilience of the method to moderate noise and camera fluctuations. Additionally, we tested the algorithm’s performance under spatter-rich conditions by artificially increasing spatter density through optical overlays and exposure manipulation. The detection accuracy remained consistent above 90%, indicating the method’s capability to isolate relevant features even in visually complex frames. These results affirm the algorithm’s suitability for real-world factory environments where lighting variability and high-density spatter events are common.

### 3.5. Spatter Formation Mechanisms

Spatter behavior in LDED is governed by fundamental melt pool dynamics, particularly Marangoni convection and vapor recoil pressure. Marangoni convection arises from surface tension gradients induced by temperature variations across the melt pool, causing fluid flow from hotter to cooler regions. Vapor recoil pressure results from intense localized vaporization at the laser–material interface, generating upward momentum that can eject molten droplets. These mechanisms collectively destabilize the melt pool and promote spatter formation. These thermal–fluid phenomena arise due to surface tension gradients and rapid vaporization under high energy input, causing instability in the melt pool and driving spatter ejection. Higher laser powers increase recoil-induced ejection, explaining the observed rise in spatter count and dispersion. Although direct thermal–flow simulation is beyond the scope of this work, we discuss the potential for the future coupling of image-based data with computational models. Additionally, the spatter features observed, particularly angle and dispersion, may indirectly reflect process irregularities such as unstable melt pool motion. Higher energy densities promote stronger recoil jets and Marangoni convection, leading to greater spatter ejection and chaotic trajectories.

## 4. Conclusions

This study reports the development of a novel image processing algorithm for extracting spatter characteristics, including count, orientation, area, and distance from the melt pool during LDED. The analysis examined the average spatter count at different laser power settings. Additionally, spatter detection was employed to assess the impact of spatter formation on deposition continuity. This study provides a method for detecting spatters, correlating them with LDED deposit quality. Future efforts will expand this framework to multi-layer builds and incorporate hybrid vision–simulation tools to enable closed-loop control. Higher-frequency CMOS cameras with different orientations will be utilized in the future to capture spatter velocity more accurately. To enhance the precision of the detection algorithm, spatter overlapping will be carefully analyzed and addressed. Further efforts will focus on distinguishing between droplet spatters and powder spatters to refine the classification process. Finally, the spatter detection algorithm will undergo rigorous validation to ensure reliability and robustness under varying process conditions. Furthermore, the detection algorithm offers immediate applicability for real-time monitoring. With low computational complexity and high accuracy, it is suitable for integration with process control systems. Future work will include multi-track and multi-layer experiments to evaluate the effect of thermal accumulation and interlayer bonding on spatter behavior. This is essential to validating the generalizability of the algorithm to full 3D builds. The use of dimensionless numbers and thermal–flow coupling simulations may also help quantify relationships between spatter behavior and melt pool conditions.

## Figures and Tables

**Table 1:** Process parameters used for deposition.

Process Parameter	Value/Range
Laser power	400–1000 Watts
Scanning speed	6 mm/s
Powder feed rate	5 g/min
Shielding gas feed rate	5 L/min

## Data Availability

The original contributions presented in this study are included in the article. Further inquiries can be directed to the corresponding author.
